# Ferroptosis and its emerging role in tumor

**DOI:** 10.52601/bpr.2021.210010

**Published:** 2021-08-31

**Authors:** Xiaoxuan Wang, Zicheng Liu, Lijuan Ma, Haijie Yu

**Affiliations:** 1 Dr Neher’s Biophysics Laboratory for Innovative Drug Discovery/State Key Laboratory of Quality Research in Chinese Medicine, Macau University of Science and Technology, Taipa, Macau, China; 2 School of Pharmacy, Macau University of Science and Technology, Macau, China

**Keywords:** Ferroptosis, Cancer, Cell death, GPX4

## Abstract

Ferroptosis is a novel form of programmed cell death characterized by iron-dependent lipid peroxidation accumulation. It is morphologically, biochemically, and genetically distinct from other known cell death, such as apoptosis, necrosis, and pyroptosis. Its regulatory mechanisms include iron metabolism, fatty acid metabolism, mitochondrial respiration, and antioxidative systems eliminating lipid peroxidation, such as glutathione synthesis, selenium-dependent glutathione peroxidase 4, and ubiquinone. The disruption of cellular redox systems causes damage to the cellular membrane leading to ferroptotic cell death. Recent studies have shown that numerous pathological diseases, like tumors, neurodegenerative disorders, and ischemia-reperfusion injury are associated with ferroptosis. As such, pharmacological regulation of ferroptosis either by activation or by suppression will provide a vast potential for treatments of relevant diseases. This review will discuss the advanced progress in ferroptosis and its regulatory mechanisms from both the antioxidative and oxidative sides. In addition, the roles of ferroptosis in various tumorigenesis, development, and therapeutic strategies will be addressed, particularly to chemotherapy and immunotherapy, as well as the discoveries from Traditional Chinese Medicine. This review will lead us to have a comprehensive understanding of the future exploration of ferroptosis and cancer therapy.

## INTRODUCTION

Cancer is the second leading cause of human death worldwide, with over 18.1 million new cancer cases and 9.6 million cancer deaths per year (Bray *et al*. 2018). Conventional radiotherapy, chemotherapy, and immunotherapy kill cancer cells by inducing cell death; however, cancer cells become resistant, resulting in multidrug resistance (MDR) in many cancer patients, and gradual treatments are not effective. As an inevitable part of the life cycle, cell death is the marker of the end of cell life. Recent studies have shown that there are other novel types of cell death in addition to apoptosis. Ferroptosis is a distinctive biological process and pathophysiological mode of programmed cell death and is mostly linked with various diseases such as tumors, neurodegenerative diseases, and ischemia/reperfusion injury. Notably, an overwhelming interest has been developed in tumor cells growth inhibition related to ferroptosis (Stockwell *et al*. 2017). It provides a new view for further understanding tumorigenesis, cancer progress, and cancer metastasis. The purposely induction of ferroptosis in specific uncontrolled tumor cells has opened up new therapeutic avenues for solving cancer clinical problems, such as drug resistance. This article summarizes the regulatory mechanisms of ferroptosis and its roles in cancer and possibly clinical practices.

## THE DISCOVERY OF FERROPTOSIS

In 2003, Dolma *et al*. discovered that a novel compound, erastin, could selectively induce a specific non-apoptotic programmed cell death in tumor cells expressing small T (ST) and NRAS^V12^ oncoproteins (Dolma *et al*. 2003). This unique cell death occurs rapidly and irreversibly without morphological changes or DNA fragmentation. Pan-caspase inhibitors cannot rescue this cell death. By using affinity chromatography, Yagoda *et al*. found that erastin acts on voltage-dependent anion channels 2 and 3 (VDACs2/3), and interferes with mitochondrial function. Large amounts of oxidizing substances are generated, eventually leading to cell death (Yagoda *et al*. 2007). RSL3 and RSL5 could also induce this type of programmed cell death, which could be reversed by applying the iron chelator, deferoxamine mesylate, and the antioxidant Vitamin E (Yang and Stockwell 2008). In fibrosarcoma cells carrying NRAS mutant, erastin increases reactive oxygen species (ROS) in the cytoplasm and plasma membrane lipids with subsequent cell detachment and death (Dixon *et al*. 2012). Iron chelators and desferrioxamine could inhibit ROS accumulation and suppress cell death. The incubation of cells with the exogenous iron ions increases ROS accumulation and cell death in the same manner as before. Therefore, Dixon *et al*. named this new type of cellular programmed death with iron-dependent ROS accumulation as ferroptosis.

## CHARACTERISTICS OF FERROPTOSIS

The morphological and biochemical characters, regulatory pathways, and genes involved in ferroptosis are different from known cell death, such as apoptosis, necrosis, autophagy, and pyroptosis (Table 1) (Galluzzi *et al*. 2018). There is an increase in membrane density, marked shrinkage of mitochondria and a decrease in mitochondrial density accompanied by a reduction or disappearance of mitochondrial cristae in ferroptosis cells (Dixon *et al*. 2012; Xie *et al*. 2016). Necrosis is associated with swelling of the cytoplasm and organelles, accompanied by the expansion of the endoplasmic reticulum and nuclear membranes, which finally leads to membrane rupture and contents discharge. Apoptosis is usually separated from adjacent cells and appears as a crumpling, rounding, and vesicular bulge rather than swelling. Besides, chromatin condensation, nuclear fragmentation, and DNA degradation are the typical characteristics of apoptosis, which lead to nuclear disruption and the formation of multiple apoptotic bodies formed by cytosolic invagination of the plasma membrane. Similar to apoptosis, DNA degradation and nuclear condensation occur when cells undergo pyroptosis, and the activation of inflammasomes leads to perforation of cell membranes, causing ions and water influx into the cells, resulting in lysis and content release (Jorgensen and Miao 2015). In terms of cargo delivery mode to the lysosome during autophagy, there are at least three distinct forms of autophagy, microautophagy, macroautophagy, and chaperone-mediated autophagy in mammalian cells. All of them are typically characterized by bilayer autophagosomes (Parzych and Klionsky 2014). Therefore, ferroptosis is quite different from other known cell death.

**Table 1:** Features of different types of cell death

Types	Ferroptosis	Apoptosis	Autophagy	Necroptosis	Pyroptosis
Morphological features	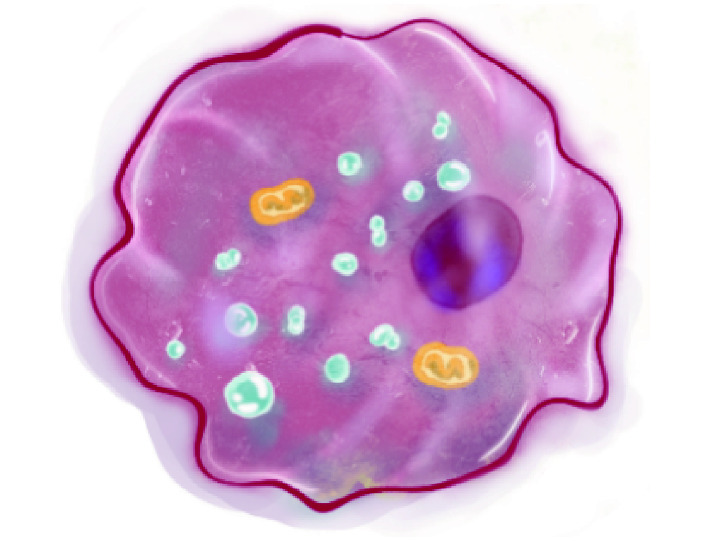	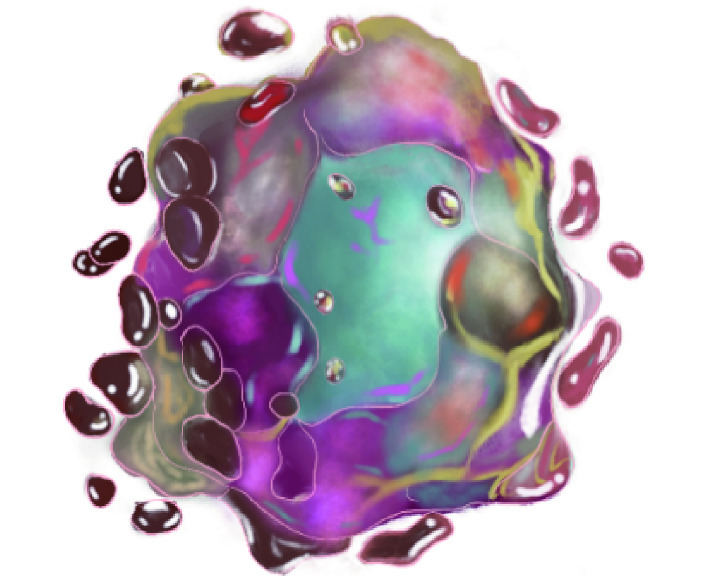	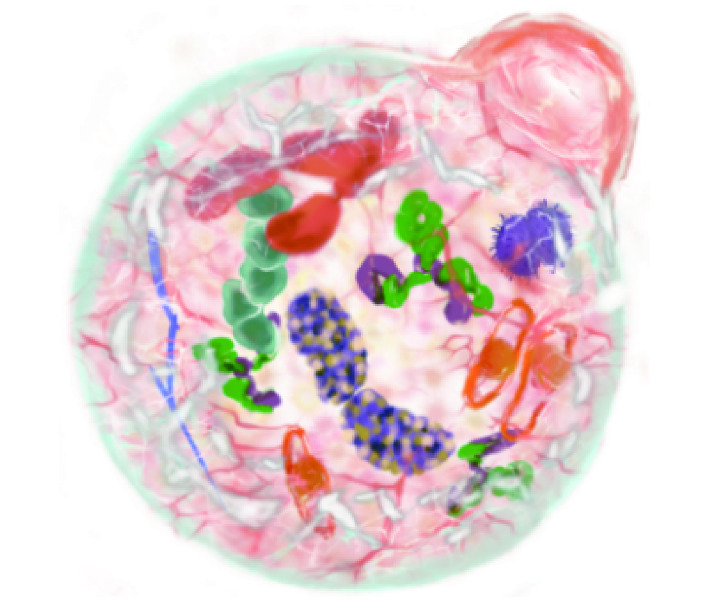	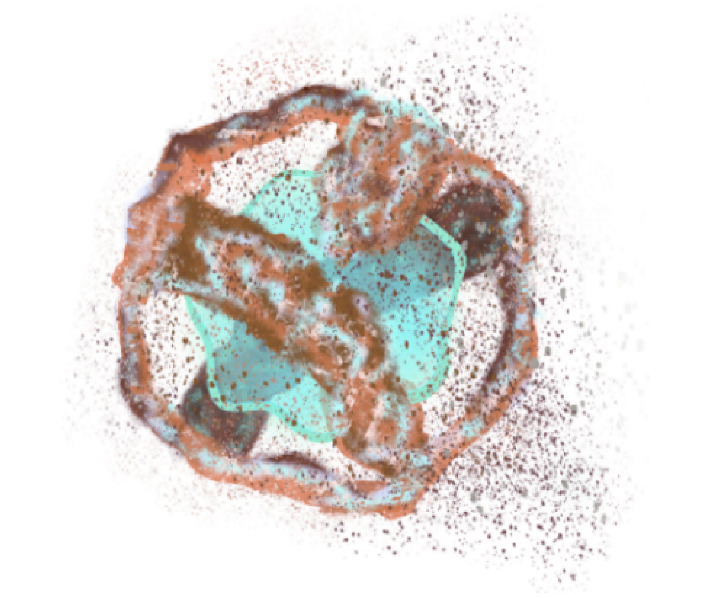	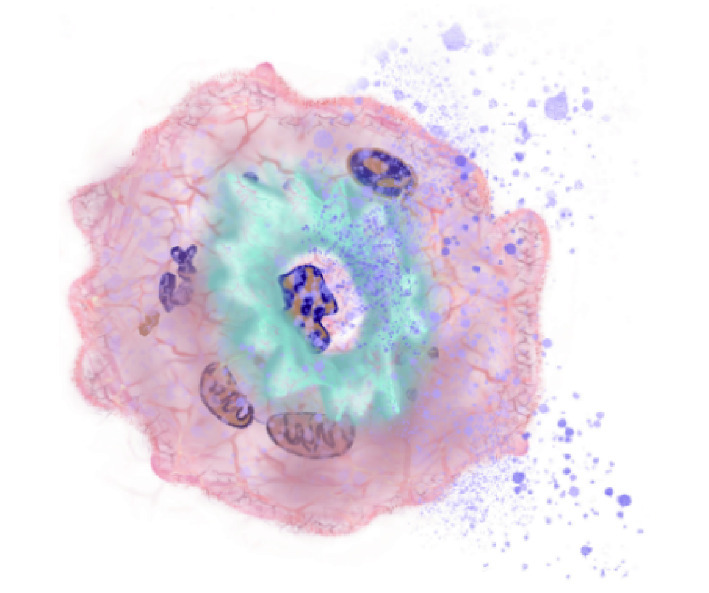
Biochemical features	Iron accumulation and lipid peroxidation	DNA fragmentation and caspase activation	Increased lysosomal activity; P62 degradation	Decreased ATP levels	Inflammatory caspase activation
Regulatory pathways	Xc^−^/GPX4; MVA; P62-Keap1-NRF2; P53/SLC7A11; ATG5-ATG7-NCOA4; P53-SAT1-ALOX15; HSPB1-TRF1; FSP1-C_O_Q10-NAD(P)H	Death receptor pathway; mitochondrion pathway; endoplasmic reticulum pathway; caspase, P53, Bcl-2 mediated signaling pathway	mTOR; Beclin-1; P53 signaling pathway	TNF-R1 and RIP1/RIP3-MLKL related signaling pathways; PKC-MAPK-AP-1 pathway; ROS-related metabolic regulation pathway	Caspase-1 and caspase-4/5/11
Key genes	GPX4; TFR1; SLC7A11; NRF2; NCOA4; P53; HSPB1; ACSL4; FSP1	Caspase; Bcl-2; Bax; P53; Fas	ATG5; ATG7; LC3; Beclin-1; DRAM3; TFEB	RIP1; RIP3	Caspase-1; IL-1β; IL-18

## REGULATORY MECHANISMS AND SIGNALING PATHWAYS OF FERROPTOSIS

**Figure 1:**
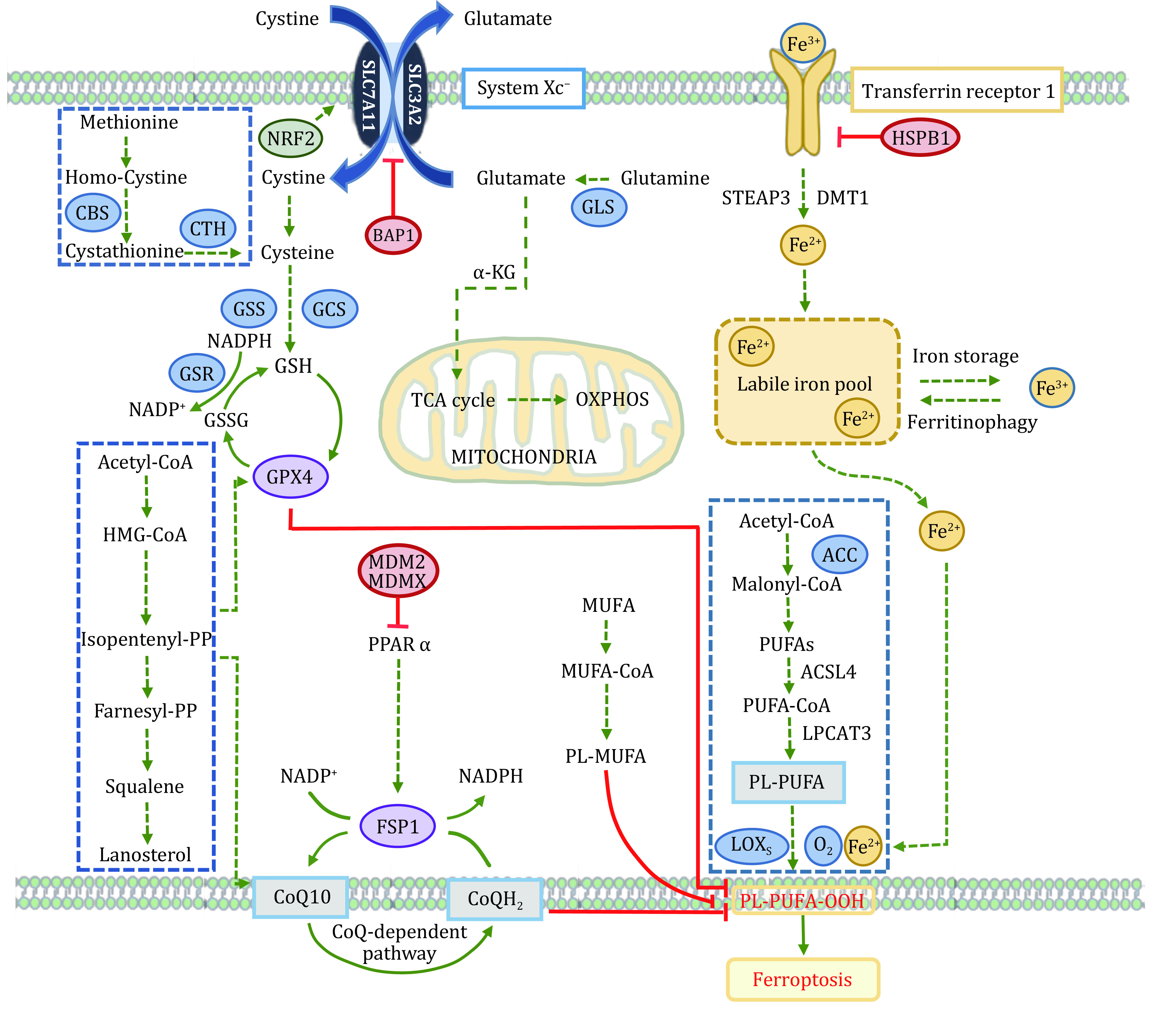
Ferroptosis molecular mechanisms and signaling pathways. Lethal iron-dependent lipid peroxidation evokes ferroptosis, which is induced due to ferroptosis-executing overwhelming and/or ferroptosis-preventing. Iron and lipid peroxidation are parts of ferroptosis-executing, while cyst(e)ine/GSH/GPX4, NAD(P)H/CoQ10/FSP1, *etc*., help cells survive from ferroptosis

Ferroptosis is a form of regulated cell death, which could balance the life-saving and death-executing processes. If the death-executing is overwhelming and (or) life-saving is defective. The death could be irreparable. Iron-dependent lipid peroxidation induces ferroptosis. Iron and lipid peroxidation are parts of death-executing, while anything fighting against iron and lipid peroxidation helps cells survive from ferroptosis. Multiple life-saving systems are involved for ferroptosis, such as cysteine/GSH/GPX4, NAD(P)H/CoQ10/FSP1, and BH_4_/GCH1/DHFR (Fig. 1).

### Cyst(e)ine/GSH/GPX4

System x_c_^−^, an indispensable cellular antioxidant system, is a heterodimer of SLC7A11 (xCT) and SLC3A2 (4F2hc and CD98hc) linked by disulfide bonds (Sato *et al*. 1999). It is ubiquitously distributed at the plasma membrane and can mediate exchange at a 1∶1 ratio between extracellular cystine and intracellular glutamate. The intracellular cystine is subsequently converted to cysteine through NADPH reduction reaction, thus promoting glutathione (GSH) synthesis (Koppula *et al*. 2018). GSH plays an essential role in regulating the ROS signal, and its level can reset the cellular redox homeostasis, including the redox situation in lipid (Kwiecien *et al*. 2014). SLC7A11 has functional diversity in regulating many pathological diseases. The stacked evidence demonstrated that SLC7A11 is upregulated in many cancers and is associated with drug resistance and patients’ poor prognosis (Lin *et al*. 2020). Suppressing SLC7A11 transporter activity or disrupting cystine homeostasis induces potent ferroptosis in many cancer cells. Microarray analysis found that P53 could inhibit System x_c_^−^ by down-regulating the expression of SLC7A11, thus affecting the activity of downstream signaling and causing ferroptosis (Jiang *et al*. 2015). Besides, several other transcription factors or tumor suppressors, such as activating transcription factor 3 (ATF3), BRCA1 associated protein-1 (BAP1), ADP-ribosylation factor (ARF), and Kelch-like ECH-associated protein 1 (KEAP1), diminish the expression or suppress the activity of SLC7A11 to induce ferroptosis in cancer cells (Fan *et al*. 2017; Roh *et al*. 2017; Wang *et al*. 2020; Zhang *et al*. 2018b). Several small-molecule compounds, including erastin, sulfasalazine, and sorafini, can inhibit SLC7A11 and block glutathione synthesis by reducing cystine uptake, leading to decreased cellular antioxidant capacity and accumulation of intracellular lipid ROS, eventually resulting in cell death accompanied by oxidative damage (Sato *et al*. 2018). Notably, under stress conditions, several proteins, such as ATF4 and NRF2, actively promote the resistance to ferroptosis by upregulation of SLC7A11. These results reveal the complexity of the SLC7A11 related signaling network (Chen *et al*. 2017; Fan *et al*. 2017).

Glutathione Peroxidase (GPx) is an essential family enzyme that catalyzes the reduction of hydrogen peroxide to water and oxygen. It is also able to convert peroxide radicals to alcohol and oxygen. GPX4 is a vital phospholipid hydroperoxidase that protects cells against ferroptosis. Its primary function is to reduce lipid peroxides (L-OOH) to their corresponding non-toxic alcohols (L-OH) by utilizing the catalytic conversion of glutathione to oxidized glutathione (Imai *et al*. 2017). As selenocysteine is an essential amino acid for the active group of GPX (Kryukov *et al*. 2003). Cells expressing a cysteine variant instead of selenocysteine are susceptible to peroxide-related ferroptosis (Ingold *et al*. 2018). Selenocysteine is required for GPX4, indicating that the mevalonate pathway (MVA) for selenocysteine synthesis is involved in ferroptotic cell death (Warner *et al*. 2000). GPX4 expression is upregulated in a variety of cancers. Kinowaki *et al*. found a strong association between GPX4 and myeloid B-cell lymphoma prognosis (Kinowaki *et al*. 2018). Notably, in some therapy-resistant cancer cells with mesenchymal cell-state contexts, inhibition of GPX4 could effectively sensitize cells to ferroptosis (Hangauer *et al*. 2017; Viswanathan *et al*. 2017). Several compounds, such as RSL compounds, ML compounds, and DPI compounds, act directly on GPX4 and inhibit its activity (Sui *et al*. 2018; Weiwer *et al*. 2012; Yang *et al*. 2014), which could be the possible drugs for further study. In general, GPX4 plays a central role in the regulation of ferroptosis. Its activity determines the ferroptosis by adjusting ROS accumulation and oxidative stress. Many targets of various ferroptosis inducers are relevant to GPX4.

### NAD(P)H/CoQ10/FSP1

Ferroptosis is evoked by the imbalance of cellular oxidation-reduction in which NAD(P)^+^ and NAD(P)H are the most critical cofactors. NAD(P)^+^ takes electrons to become NAD(P)H by coupling with metabolic processes. Numerous critical biologically essential reactions such as ATP production, catabolism, anabolism, and cellular redox regulation happen through these electrons transferring. Besides the axis SLC7A11-GSH-GPX4, NAD(P)H-FSP1-CoQ signaling pathway is validated as another important ferroptosis defense system. The associated factor apoptosis inducing factor mitochondria associated 2 (AIF-M2) is a potent anti-ferroptosis factor (Bersuker *et al*. 2019; Doll *et al*. 2019). A previous study showed that AIF-M2 contributed to apoptosis, and its gene expression is a p53 responsive one (Gong *et al*. 2007). Subsequent studies revealed that AIF-M2 is identified ferroptosis suppressor protein-1 (FSP1), which can inhibit ferroptosis induced by GPX4 depletion, while its knockdown sensitizes the cells to ferroptosis inducers. FSP1 protein shares homology to NADH oxidoreductases, which confers its functions to reduce ubiquinone (CoQ10) to ubiquinol (CoQ10H2) and to regenerate CoQ10 through catalyses of NAD(P)H to NAD(P). Ubiquinol acts as an antioxidant trapping lipid peroxyl radicals, so FSP1 can halt the propagation of lipid peroxides and stop the ferroptosis. However, another study shows the ubiquinol-independent mechanisms underlying the blockage of FSP1-mediated ferroptosis, which endosomal sorting complexes required for transport (ESCRT)-III is required (Dai *et al*. 2020b). These results reveal that the ferroptosis and ferroptosis resistance are correlated with FSP1. Various factors are regulating the expression of FSP1, such as cAMP-response-element-binding protein (CREB) (Nguyen *et al*. 2020), mouse double minute 2 homolog/murine double minute X (MDM2/MDMX) complex (Venkatesh *et al*. 2020). Therefore, FSP1 regulators have great potential to overcome ferroptosis resistance in cancer treatments. Interestingly, mitochondrial GPX4 operates with mitochondrial dihydroorotate dehydrogenase (DHODH) to inhibit ferroptosis by reducing CoQ10 to CoQ10H2, the independent reduction pathway of cytosolic GPX4 and FSP1. In cancer treatment, the study suggests a therapeutic strategy for targeting ferroptosis in specific local organelles, such as mitochondria (Mao *et al*. 2021).

### BH4/GCH1/DHFR

The recently established BH4/GCH1/DHFR7 axis is another paralleled signal pathway that diminishes the ferroptosis. Tetrahydrobiopterin (BH4) biosynthesis is identified through genome-wide screens upon Cyst(e)ine/GSH/GPX4 inhibition (Kraft *et al*. 2020; Soula *et al*. 2020). BH4 is a robust antioxidant trapping lipid radical and promotes CoQH2 regeneration against lipid peroxidation and ferroptosis. The enzymes in the BH4 pathway, such as the GTP cyclohydrolase-1 (GCH1), the rate-limiting enzyme for synthesis BH4, and dihydrofolate reductase (DHFR), the enzyme for BH4 regeneration, are indispensable for the protection of cells counteracting the effects of ferroptosis.

### PUFA metabolic pathway and lipid peroxidation

In the cell membrane, polyunsaturated fatty acid (PUFA) is a critical component and is the molecular basis for the fluidity and deformability of cell membranes (Yehuda *et al*. 2002). It is generated by successive biochemical reactions of elongation and desaturation through multiple metabolic enzymes. Manipulation of PUFA production and degradation has a significant impact on ferroptotic cell death because PUFA is the major substrates for lipid peroxidation due to its double bond vulnerability to radical-mediated oxidation. Suppression of Acetyl-CoA carboxylase (ACC) pharmacologically or genetically significantly blocks ferroptosis response because it catalyzes the synthesis of malonyl-CoA required for PUFA (Lee *et al*. 2020a; Shimada *et al*. 2016). Free PUFA is not the main radical target (Kagan *et al*. 2017). It becomes a target after being esterified and incorporated into plasma membrane by acyl-coenzyme A synthetase long chain family member 4 (ACSL4) and lysophosphatidylcholine acyltransferase 3 (LPCAT3) (Yang *et al*. 2016). Accordingly, the knockdown of these two genes in cells decreased PUFA synthesis and inhibited cellular ferroptosis (Dixon *et al*. 2015; Doll *et al*. 2017). During ferroptosis, plasma membrane PUFA is attacked by free radicals and oxidized to phospholipid hydroperoxide (PLOOH). However, the exact mechanism details are not fully understood. Is it an enzymatic catalyzation or enzyme-independent reaction or combination? Lipoxygenases (ALOXs), particularly ALOX15, have been reported to peroxidize PUFA and eventually induce cellular ferroptosis (Wenzel *et al*. 2017). In contrast, other studies reveal the opposite results that KO *Alox15* does not rescue the ferroptosis induced by GPX4 depletion (Brutsch *et al*. 2015; Shah *et al*. 2018). Recent studies show that regulation of PUFA abundance at the plasma membrane can change the response to ferroptosis. n-3 but also remarkably n-6 polyunsaturated FA selectively induced ferroptosis in cancer cells under ambient acidosis (Dierge *et al*. 2021). Displacement of PUFA with monounsaturated fatty acids (MUFA) render cells resistant to ferroptosis. Besides, two key enzymes, stearoyl coenzyme A desaturase (SCD) and acyl-coenzyme A synthetase long chain family member 3 (ACSL3), regulate the MUFA abundance at the plasma membrane and change the sensitivity to ferroptosis, again pointing to the same point that MUFA is a critical target for ferroptosis (Magtanong *et al*. 2019; Tesfay *et al*. 2019).

### Iron metabolism

Iron is an essential trace element in the body, whose metabolism refers to absorption, storage, and utilization. The abnormalities of iron distribution and metabolism in the body will affect the body's normal physiological processes. Most of the iron intake from food after intestinal absorption is Fe^3+^. It binds to transferrin (TF) to form the TF-Fe^3+^ complex, which is taken up by TF receptors (TFR1) at the plasma membrane (Fleming *et al*. 1998). The TF-Fe^3+^/TFR1 complex undergoes internalization through clathrin-mediated endocytosis and further degradation in endo-lysosome, where low pH aids to release the Fe^3+^. Then Fe^3+^ is reduced to Fe^2+^ by the proteins of the six-transmembrane epithelial antigen of the prostate (STEAP) family (Hvidberg *et al*. 2005; Ohgami *et al*. 2006), so that Fe^2+^ can be transported to cytoplasm across the endosomal membrane through the divalent metal-ion transporter 1 (DMT1) (Yanatori and Kishi 2019). Fe^2+^ can be further exported to the extracellular space by ferroportin 1 (FPN1). Circulating TF only binds Fe^3+^, so iron oxidation is necessary. Ceruloplasmin proteins (Cp) oxidizes Fe^2+^ to Fe^3+^ and help iron release from FPN1 (Hellman and Gitlin 2002). If iron is not utilized immediately, it is stored within ferritin and hemosiderin to maintaining iron homeostasis (Frazer and Anderson 2014). During ferroptosis, iron actively generates radicals and initiates lipid peroxidation, probably via Fenton chemistry reaction and/or via participation in specific enzymatic reactions. Free iron sensitizes cells to ferroptosis, which means anything associated with cellular iron import, storage, export, and another metabolism may be involved in ferroptosis. Knockdown the *IREB2*, the core regulator of iron metabolism, renders the cells resistant to erastin-induce ferroptosis (Dixon *et al*. 2012). Blocking the iron import by iron chelators, iron-free apo-transferrin, or silencing *TFRC,* the gene encoding TFR1, can rescue the cell from ferroptosis (Gao *et al*. 2015). In addition, depletion of FPN1 (Chen *et al*. 2020b) or ceruloplasmin (Shang *et al*. 2020) increases the sensitivity of cells to ferroptosis. Hemoglobin oxygenase (HO-1) can accelerate erastin-induced ferroptosis by increasing the labile iron release from heme (Kwon *et al*. 2015), showing that the cellular heme pool contributes to the intracellular iron store and correlates to ferroptosis. Besides, Zip14 (Slc39a14) mediates non-transferrin-bound iron uptake. Depletion of Zip14 significantly reduced ferroptosis-mediated liver fibrosis in the pathological condition (Yu *et al*. 2020b).

Collectively, multiple cellular processes and signaling pathways have been linked to ferroptosis. Furthermore, cells are continuously receiving many extracellular signals from the microenvironment and/or circulation system. If counting the signal of ferroptotic cells, either released actively or leaked passively (Efimova *et al*. 2020; Wen *et al*. 2019; Yu *et al*. 2020a), there will be a great unknown to be explored. Further investigations are needed to have a complete understanding of ferroptosis and its impacts on body homeostasis, which can be applied in disease-related therapeutic strategies.

## ROLE OF FERROPTOSIS IN TUMORS

**Figure 2:**
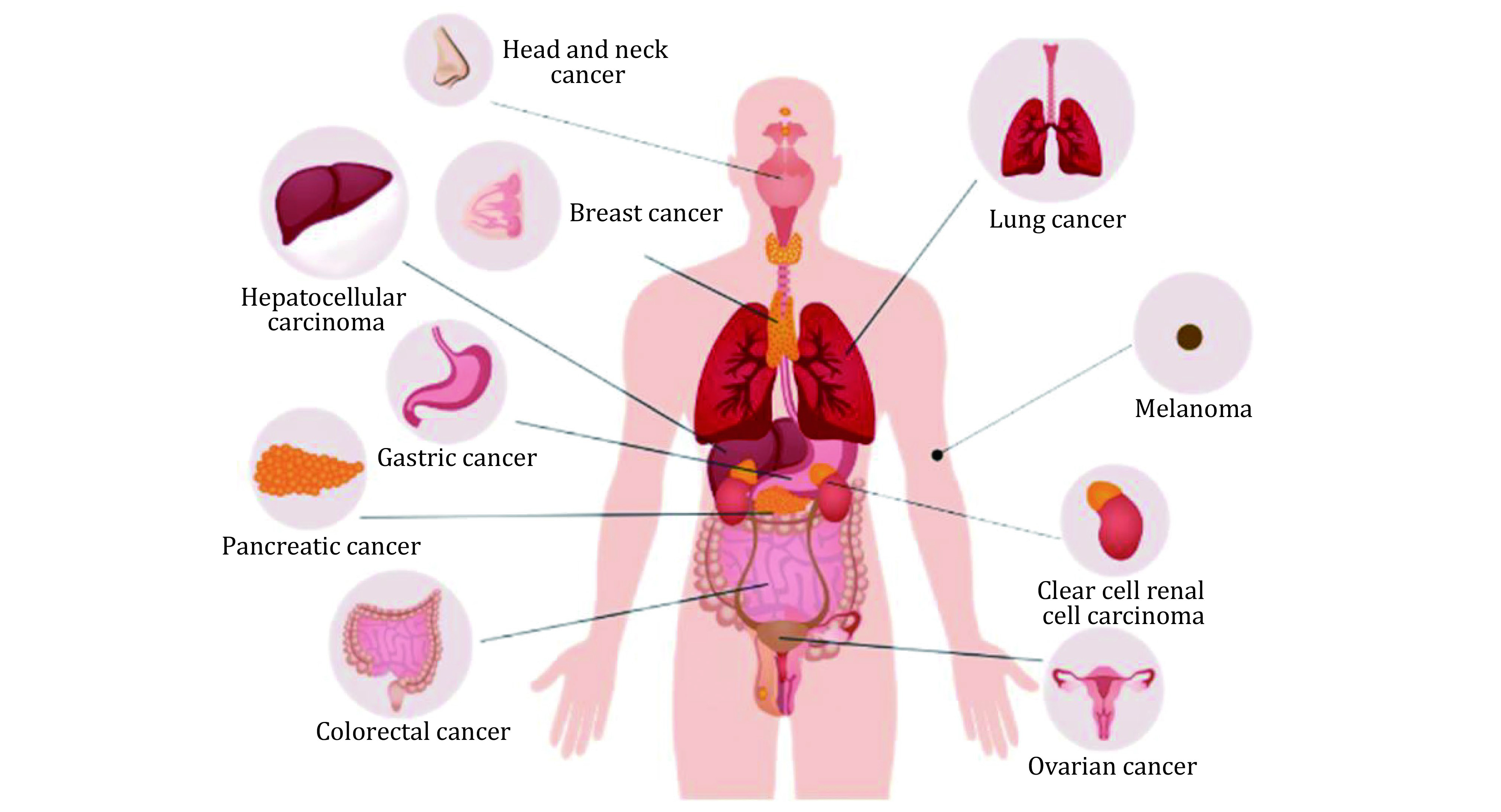
The possible actions of ferroptosis on various cancers including digestive system, respiratory system, renal and urinary system, endocrine system, reproductive system and integumentary system

As the ferroptosis mechanisms are continually being studied, more and more researches show that ferroptosis is closely associated with tumors, ischemia-reperfusion injury, and neurodegenerative diseases. In the following section, we will focus on the emerging roles of ferroptosis in various cancers, which have been broadly studied (Fig. 2). Besides, in cancer therapeutic studies, ferroptosis inducers are effective tumors suppressors, indicating their great potential for further drug development (Jiang *et al*. 2021).

### Head and neck cancer

Head and neck cancer (HNC) mainly include tumors of the mouth, nose, and pharynx. RSL3 and ML-162, inhibitors of GPX4, have been shown to induce ferroptosis in HNC cells (Shin *et al*. 2018). Artesunate (ART) can also induce ferroptosis in HNC cells through GSH depletion and ROS accumulation (Roh *et al*. 2017). However, the cisplatin-resistant HNC cell is not sensitive to ferroptosis inducers and ART due to upregulated Nrf2–antioxidant response element (ARE) signal. The resistance can be eliminated by blocking the Nrf2-ARE pathway, thereby recover the sensitivity to ferroptosis. CISD2 is also critical for anti-oxidative effects in head and neck tumor cells. Functional disruptions of CISD2 cause the increases of the mitochondrial iron and lipid ROS accumulation, thereby inducing cell ferroptosis (Kim *et al*. 2018). Recent studies showed that inhibition of glutaredoxin 5 (GLRX5) could promote lipid peroxidation and increase intracellular free iron by interfering with the iron metabolism signal, including increasing transferrin receptors and decreasing ferritin (Lee *et al*. 2020b).

### Breast cancer

Breast cancer is one of the most deadly types of cancer in women, especially in triple-negative breast cancer (TNBC), accounting for approximately 10%–20% of all breast cancer cases (O’Toole *et al*. 2013). Timmerman *et al*. revealed that a subset of triple-negative breast cancer cell lines is highly dependent on xCT to obtain environmental cysteine. xCT inhibitor inhibits tumor growth by promoting ferroptosis (Timmerman *et al*. 2013). Another study showed that xCT could interact with mucoprotein-1-C (MUC1-C) and CD44 variant (CD44v) to keep stabilized on the plasma membrane surface, so the GSH levels can be sustained to prevent ferroptosis in TNBC cells (Hasegawa *et al*. 2016). Lysosomal disrupting agent siramexin and tyrosine kinase inhibitor lapatinib can interfere with ROS signal and the iron homeostasis, leading to ferroptosis in the breast cancer cells. Removal of the iron by iron chelator or overexpression of ferroprotein-1 can suppress the effects of Silamexine and Lapatinib (Ma *et al*. 2016). GPX4 inhibition enhanced the antitumor effect of gefitinib by promoting ferroptosis, designating a promising strategy to deal with drug-resistant problems (Song *et al*. 2020).

### Lung cancer

Lung cancer is the leading cause of cancer death. It is broadly divided into small cell carcinoma (SCLC 20%–25% of cases) and non-small cell lung cancer (NSCLC 70%–80% of cases). Lung adenocarcinoma has abundant expressions of the iron-sulfur cluster biosynthetic enzyme NFS1, harboring high oxygen tension resistance and protecting cells from ferroptosis. Limiting NFS1 action and reducing the glutathione biosynthesis can drive cells to ferroptosis, which can be used as an advanced method for lung cancer treatments (Alvarez *et al*. 2017). Another study showed that lung adenocarcinoma carrying mutations in STK11 and KEAP1 has significant upregulation of ferroptosis-protective proteins, such as SCD and aldo-keto-reductase-1C. Inhibition of SCD1 augments the effects of ferroptosis inducers, indicating that the SCD1 is a likely candidate for targeting the specific mutant induced lung cancer (Wohlhieter *et al*. 2020).

### Gastric cancer

In gastric cancer cells, erastin could also induce ferroptosis, in which Type I cysteine dioxygenase (CDO1) plays an essential role in the process (Hao *et al*. 2017). CDO1 competitively binds to cysteine, limiting GSH synthesis and ultimately leading to ferroptosis. Inhibition of COD1 activity prevents ROS production, maintains the mitochondrial functional stability, and decreases lipid peroxidation, eventually inhibiting ferroptosis. Besides the tumor cells, tumor-associated fibroblasts (TAFs) promote tumor growth, development, even drug resistance acquisition. Exosomal miR-522 secreted by TAFs prevents the lipid ROS accumulation and ferroptosis by inhibiting the ALOX15 activity, which explains the abnormality of ALOX15 in gastric cancer (Zhang *et al*. 2020).

### Pancreatic cancer

Pancreatic cancer is a highly lethal tumor with an average five-year survival rate of less than 10% (Siegel *et al*. 2016). Artesunate (ART) can specifically induce intracellular ROS elevation, resulting in ferroptosis in pancreatic ductal adenocarcinoma cells (Eling *et al*. 2015). Besides, the composition of Cotylenin A (CN-A), a potent inducer of differentiation, and phenethyl isothiocyanate (PEITC), a ROS inducer, can also induce cellular ROS production and lead to cell death of ferroptosis (Kasukabe *et al*. 2016). Recently, Badgley *et al*. found that inhibiting cysteine uptake induces ferroptosis in pancreatic ductal adenocarcinoma. *In vivo* studies have shown that deletion of SLC7A11 and administration of cyst(e)inase inhibited PDAC growth by ferroptosis induction (Badgley *et al*. 2020). The results bring new approaches and ideas to the clinical dilemma of pancreatic cancer treatment.

### Hepatocellular carcinoma

Hepatocellular carcinoma (HCC) is a highly heterogeneous disease with a low survival rate. It also has solid adverse reactions and drug resistance to some first-line drugs, such as sorafenib (Nie *et al*. 2018). In the HCC cells, sorafenib-induced ferroptosis is associated with the Sigma-1 receptor (S1R) regulation and NRF2 mediated peroxidation increase (Bai *et al*. 2019). During HCC pathological process, retinoblastoma protein (pRb) is an essential indicator, and loss of Rb function in HCC cells promotes ferroptosis induced by sorafenib and results in tumor regression (Louandre *et al*. 2015). Additionally, nanoparticles loaded with natural omega-3 fatty acid and docosahexaenoic acid (LDL-DHA) selectively inhibit the HCC cell growth and suppresses the liver tumor due to the GSH depletion, GPX4 inactivation, and lipid peroxidation, eventually causing ferroptosis (Ou *et al*. 2017).

### Colorectal cancer

Colorectal cancer is currently the third most common cancer in humans, also the second most common cancer mortality (Bray *et al*. 2018). As the central regulator, TP53 controls cell proliferation, metabolism, death and differentiation. The functions of TP53 are also complex in the ferroptosis regulation (Khoo *et al*. 2014). p53 represses the SLC7A11 transcription by direct binding to the SLC7A11 promoter region or by impacts on ALOX proteins to induce ferroptosis (Chu *et al*. 2019; Jiang *et al*. 2015; Ou *et al*. 2016). However, it also sustains the GSH level by p21 upregulation to prevent ferroptosis (Tarangelo *et al*. 2018). In colorectal cancer cells, p53 could constrain erastin-stimulated ferroptosis by inhibiting dipeptidyl peptidase-4 (DPP4) in the transcription-independent manner (Xie *et al*. 2017). The interaction between p53 and ferroptosis is correlated to the cellular context and specific induction, which makes it gain more attention and investigation.

### Renal cell carcinoma

Renal cell carcinoma (RCC) is a devastating disease with significant metabolic reprogramming and resistance to conventional therapies. Investigation reveals that clear cell renal cell carcinoma (ccRCC) is highly vulnerable to ferroptosis due to its disordered GSH/GPX pathway (Miess *et al*. 2018). Recently, Yang *et al*. found that transcriptional coactivator with PDZ-binding motif (TAZ) regulates the expression of Epithelial Membrane Protein 1(EMP1), which induces the NADPH Oxidase 4(NOX4) to generates ROS (Yang *et al*. 2019), eventually kills the cell. These results imply that ferroptosis induction in RCC cells may be a more practical alternative for renal cancer treatments.

### Melanoma

In studies on melanoma, the progressive differentiation subtypes of melanoma are closely related to the ferroptosis pathway (Tsoi *et al*. 2018). There are several studies about micro RNA on ferroptosis modulation. miR-137 negatively regulates ferroptosis by directly acting on the glutamine transporter protein SLC1A5 (Luo *et al*. 2018), while miR-9 regulates ferroptosis by aiming to glutamic-oxaloacetic transaminase GOT1 (Zhang *et al*. 2018a). In mitochondria, inhibition of mitochondrial complex I increase ROS accumulation and eventually leads to cell death involved with ferroptosis and necroptosis in melanoma (Basit *et al*. 2017). Notably, erastin binds to mitochondrial voltage-dependent anion channels VDAC2 and VDAC3 at the outer mitochondrial membrane, resulting in the Nedd4-dependent degradation to maintain cellular homeostasis (Yang *et al*. 2020). Also, PEG-coated silica nanoparticles can induce ferroptosis in melanoma cells, which provides a new approach to treating melanoma (Kim *et al*. 2016).

### Ovarian cancer

Ovarian cancer is one of the top causes of cancer-related deaths among women (Siegel *et al*. 2019). ART can elevate ROS in ovarian cancer cells, leading to cellular ferroptosis (Greenshields *et al*. 2017). High-grade serous ovarian cancer (HGSOC) is a common subtype of malignant ovarian cancer. In HGSOC cells from patients, there is a disturbance of iron metabolism characterized by increased iron uptake and accumulation. Increased TFR1 accompanied by decreased expression of the iron ion pump and FPN disrupts the iron metabolism homeostasis, which accelerates the tumor growth and metastasis (Basuli *et al*. 2017). Reestablishment of the iron metabolism will render ovarian cancer tumors more sensitive to the inducer of ferroptosis. It will help researchers for further therapeutical study.

## FERROPTOSIS AND TUMOR TREATMENTS

### Ferroptosis and drug resistance in the tumor

Drug resistance is a complex process with multiple causes and is a significant hindrance limiting the recovery of patients during chemotherapy treatment and other therapeutic methods. Early multi-drug chemotherapy using drugs with different mechanisms is required to avoid drug resistance (Crofton 1959). This strategy is followed by chemotherapy with different drug dose intensities and modification of chemotherapeutic drug intervals to combat chemotherapy resistance and overcome tumor relapse (Citron *et al*. 2003; Hryniuk and Bush 1984). In addition to the previously mentioned drug resistance of sorafenib and lapatinib in hepatocellular carcinoma and breast cancer, platinum drug resistance is also referred to as a common clinical problem. Platinum drugs are clinical first-line chemotherapeutic agents, including cisplatin, cathode platinum, and oxaliplatin. Platinum drugs cause tumor cell death through DNA damage or cellular stress by lighting the death-signal fire (Wong 2011), in which elevating intracellular ROS by promoting oxidative production and/or by dampening antioxidant activity might be the critical reason(s) (Liou and Storz 2010). The high ROS level reprograms tumor metabolism or vice versa, affecting the tumor microenvironment. While tumor cells continuously adapt to the altered internal and external environment with high ROS, therefore gradually acquire drug resistance from surviving. Apoptosis and ferroptosis coincided with many tumor cells responding to cisplatin (Guo *et al*. 2018; Li *et al*. 2020). The known mechanisms of chemotherapeutic resistance are mostly related to platinum drug-induced apoptosis. Recently a few studies involved in ferroptosis were published. For example, the applications of erastin on cisplatin-resistant ovarian cancer cells resulted in the increased sensitivity to cisplatin (Sato *et al*. 2018). In HNC cells that developed cisplatin resistance, erastin treatment could reverse the resistance state (Roh *et al*. 2016). Erastin could enhance various chemotherapeutic agents' efficacy, including cisplatin, doxorubicin and cytarabine (Roh *et al*. 2017). As a completely different death regulatory mechanism for cancer cells with high ROS levels, ferroptosis opens up new therapeutic windows for multidrug resistance in cancer chemotherapy.

### Ferroptosis and tumor immunotherapy

Anticancer is the combination of tumor-killing activity of the immune system and tumor intrinsic dying. One of the most impactful anticancer therapies is the immune checkpoint blockade therapy, which boosts the killing activity of CD8+ T cells. However, only a fraction of cancer patients respond to the checkpoint blocker. In terms of ferroptosis in immunotherapy, the emerging evidence shows that CD8+ T cells kill the tumor cell by lipid peroxidation-dependent ferroptosis (Zou *et al*. 2019). INF-γ secreted from CD8+ cells is the critical cytokine. It inhibits the expression of System x_c_^−^ of tumor cells and blocks cystine uptake, causing the excessive lipid peroxidation accumulation specific for ferroptosis. Combining a ferroptosis trigger and an immune checkpoint inhibitor produces a more robust immune response against the tumor. However, the effects of ferroptosis triggering application should be considered on the immune system itself. T cell proliferation is exquisitely dependent on xCT expression in culture. Interestingly, xCT is not strictly required for T cell proliferation and functional responses to tumors *in vivo* (Arensman *et al*. 2019). The functions of GPX4 are indispensable for immune response because GPX4 functional depletion attenuates the stimulator-of-interferon genes (STING) activation (Jia *et al*. 2020), CD8+ and CD4+ T cells lacking Gpx4 failed to protect from virus and parasite infections (Matsushita *et al*. 2015). Besides, immune cells receive the signaling messages from ferroptotic tumor cells, such as exosomes containing KRAS or DAMP molecules (Dai *et al*. 2020a; Wen *et al*. 2019). The immune system may adjust to internal or trigger external ferroptosis in various tissues in specific contexts, resulting in a complex phenotype.

### Ferroptosis and tumor treatment with Chinese Medicine

China has a long history of using natural products, and her riches in compound libraries of Traditional Chinese Medicine (TCM) resources provide tremendous help in the search for ferroptosis inducers and inhibitors. Artemisinin, first discovered by a Chinese scientist Youyou Tu, is an active constituent of TCM with significant malaria inhibitory properties. The derivative artesunate can increase the ROS and inhibit the proliferation of ovarian cancer cells partially through ferroptosis (Greenshields *et al*. 2017). Dihydroartemisinin can induce apoptosis and ferroptosis in head and neck cancer cells (Lin *et al*. 2016). Erianin, a natural product isolated from *Dendrobium chrysotoxum*, elicits its anticancer activity through ferroptosis in lung cancer cells (Chen *et al*. 2020a). Piperlongumine (PL) is a natural product from long pepper. It also suppresses pancreatic cancer cells by induction of ferroptosis (Yamaguchi *et al*. 2018). Besides the synergistic inhibition of active compounds from TCM. Many other natural products have a protective role in ferroptosis. As a 12/15-lipoxygenase inhibitor, Baicalein reverses the RSL3-induced ferroptosis in acute lymphoblastic leukemia (ALL) cells (Probst *et al*. 2017). Puerarin could inhibit lipid peroxidation and iron overload in cardiomyocytes and prevent cells from ferroptosis (Liu *et al*. 2018). Besides, isothiocyanates from cruciferous species and many other natural active ingredients with antioxidant properties may be potential agents in regulating ferroptosis. Therefore, TCM can significantly prevent lipid-peroxidation-related diseases, such as cancer, heart failure, or neurodegeneration. Therefore, TCM has a broad application prospect.

## PROSPECTS

Ferroptosis has been proposed only for a few years. With the constant investigation, more and more physiopathological processes are involved in ferroptosis, significantly different types of tumor cells are associated with ferroptosis. However, the underlying mechanism is not completely clear, and there are still many questions to be further explored. For example, the relationship between different cell death mechanisms, such as apoptosis and ferroptosis, is unknown when combined with diverse anticancer agents. Current FDA-approved ferroptosis inducing agents, such as altretamine (GPX4 inhibitor) and sorafenib (system xc- inhibitor) (Woo *et al*. 2015; Yamaguchi *et al*. 2018), have significant clinical potential. However, the potential side effects associated with these small molecule drugs during oncology treatment are still unclear. More and more efforts are being made to develop ferroptosis inducers; for example, the inhibitors that act on GPX4 via a reactive alkyl chloride moiety have poor selectivity and pharmacokinetic properties (Eaton *et al*. 2020). Further development of covalent inhibitors or nano preparation technologies can be undertaken to improve the targeting and pharmacokinetics. Another consideration is from the lethal phenotype in adult mice after genetic deletion of GPX4. It will narrow the therapeutic avenue for GPX4 inhibitors and bring more challenges (Friedmann Angeli *et al*. 2014; Yoo *et al*. 2012). Furthermore, there are complex networks of genetic mutations in tumor cells, and how to assess the genetic selectivity of antitumor drugs based on ferroptotic mechanism remains to be investigated thoroughly. In summary, the comprehensive mechanisms of ferroptosis need more clarification so that the complete understanding of ferroptosis could underpin future oncology research, which will support the therapeutic drug development for cancers.

## Conflict of interest

Xiaoxuan Wang, Zicheng Liu, Lijuan Ma and Haijie Yu declare that they have no conflict of interest.
